# Acoustic Emission Source Location Using Finite Element Generated Delta-T Mapping

**DOI:** 10.3390/s22072493

**Published:** 2022-03-24

**Authors:** Han Yang, Bin Wang, Stephen Grigg, Ling Zhu, Dandan Liu, Ryan Marks

**Affiliations:** 1 TWI Ltd., Granta Park, Great Abington, Cambridge CB21 6AL, UK; stephen.grigg@twi.co.uk (S.G.); dandan.liu@brunel.ac.uk (D.L.); ryan.marks@twi.co.uk (R.M.); 2 Department of Mechanical and Aerospace Engineering, Brunel University London, Uxbridge UB8 3PH, UK; bin.wang@brunel.ac.uk; 3 Key Laboratory of High Performance Ship Technology (Wuhan University of Technology), Ministry of Education, Wuhan 430063, China; zl79111@hotmail.com; 4 School of Naval Architecture, Ocean and Energy Power Engineering, Wuhan University of Technology, Wuhan 430063, China

**Keywords:** Acoustic Emission, Non-Destructive Evaluation, Structural Health Monitoring, delta-T mapping, finite element, Hsu-Nielsen sources, source location, complex plate

## Abstract

One of the most significant benefits of Acoustic Emission (AE) testing over other Non-Destructive Evaluation (NDE) techniques lies in its damage location capability over a wide area. The delta-T mapping technique developed by researchers has been shown to enable AE source location to a high level of accuracy in complex structures. However, the time-consuming and laborious data training process of the delta-T mapping technique has prevented this technique from large-scale application on large complex structures. In order to solve this problem, a Finite Element (FE) method was applied to model training data for localization of experimental AE events on a complex plate. Firstly, the FE model was validated through demonstrating consistency between simulated data and the experimental data in the study of Hsu-Nielsen (H-N) sources on a simple plate. Then, the FE model with the same parameters was applied to a planar location problem on a complex plate. It has been demonstrated that FE generated delta-T mapping data can achieve a reasonable degree of source location accuracy with an average error of 3.88 mm whilst decreasing the time and effort required for manually collecting and processing the training data.

## 1. Introduction

Ageing-induced cracks, human-caused hazards and natural disasters such as earthquakes or hurricanes can result in the deterioration of in-service metallic structures and loss of structural integrity in the oil and gas industry [1]. Without appropriate remedial measures, structural integrity is likely to reduce further with potential to lead to catastrophic damage with risks of loss of lives and capital. In order to eliminate unplanned shutdowns and reduce inspection and maintenance costs, there is a need for a proactive monitoring approach to assess the existing structural condition and facilitate safe operation. As a method of monitoring the structural behaviour, evaluating structural performance and identifying damage at an early stage, Structural Health Monitoring (SHM) systems such as systems which include strain and vibration monitoring are gaining significant interest and increased application. Recent industrial studies suggest that detecting damage at an early stage can reduce costs and increase the life expectancy of a structure [2]. Therefore, a practical and cost-efficient SHM method that can identify micro and nano defects at an early stage and continually assess the structural health is desirable to ensure structures operate safely and efficiently throughout the service life. Adaptation of Non-Destructive Evaluation (NDE) techniques for SHM is gathering significant interest because of the ability to provide quantitative information on defects based on measured physical parameters [3]. Inspection of large-scale complex structures using NDE techniques such as X-ray and ultrasonic techniques can be laborious and expensive. However, if the damage location can be identified accurately in advance by one of the NDE techniques, i.e. the Acoustic Emission (AE) measurement technique, only the area of concern needs to be inspected. AE is defined as the transient elastic energy released by a material when it experiences a change of deformation in its structure [4]. As a passive technique, only active defects can be detected by AE testing.

Difficulties in applying the most commonly used source location method within the field of AE, Time of Arrival (TOA) technique, for locating active defects in complex structures were reported by Baxter et al. [5] and Pearson et al. [6]. The main assumptions of constant wave speed and unobstructed wave path between the AE source and sensor in TOA technique were not met due to the inhomogeneity of certain materials such as composites and the complexity of the real structures that contain features such as welds, holes and thickness. In order to overcome these difficulties, the delta-T mapping technique was developed by Baxter et al. [5]. This has been shown to improve AE source location accuracy in complex structures. In this technique, a training grid is placed on an area of special interest. The generation of a well-established and widely used artificial source of AE, Hsu-Nielsen (H-N) source [7], at each node of the grid can provide arrival time data of waves for each sensor. The difference in arrival time of waves (or delta-T) for each sensor pair can be found at each point; these data can be employed to generate maps. Once real test data has been collected, the actual delta-Ts can be used to identity a line with same and equal delta-T in corresponding map. By overlaying all the resulting maps, the intersections of all lines should converge into one point, which is the most likely location of a real AE event. Due to the good performance of this technique, it was adopted in further lab-based studies by Eaton et al. [8], Pearson et al. [6] and Grigg et al. [9]. Researchers have also improved localisation by utilising the minimum difference method [10] and generalized regression neural network [11] for identifying the source location from the delta-T map. The details of delta-T mapping technique can be found in Baxter et al. [5]. However, there is a disadvantage to this technique in that the manual collection and processing of training data requires significant time and human input. This lengthy and time-consuming process has prevented this technique from broad applications on large complex structures.

To overcome these problems, Marks [12] used Local Interaction Simulation Approach (LISA) to train the delta-T maps and locate real damage events on a plane of a complex structure. Results of fatigue crack event locations using simulated training data were compared to those from experimentally acquired training data. Similarity in the accuracy of the results of the two approaches demonstrated great potential for the use of modelling data to locate real AE events. The commercially available package based on LISA, cuLISA3D, bears high computational efficiency and thus has a short runtime (for example, running a 370 mm × 200 mm × 3 mm model takes approximately 3 min [12]). However, the application of cuLISA3D was very limited. The reported disadvantages of cuLISA3D include lacking the ability to define irregular mesh sizes and to model anisotropic materials such as composites [12]. As the Finite Element (FE) method allows for easier modelling of complex geometrical structures with anisotropic materials properties, it was employed in this study to produce simulated data required for delta-T mapping. Some studies [13,14,15,16,17,18] for simulation of AE signals from H-N sources using FE method can be found. A 2-D (two-dimensional) Dynamic Finite Element Method (DFEM) was firstly proposed by Gary and Hamstad [13] and extended to a 3-D (three-dimensional) DFEM [14] to simulate AE waveforms generated by an H-N source in thin plates. A step force was used in 2-D and 3-D models to excite the AE waves. Later, the lead break in an H-N source was also simulated using an abrupt change of boundary conditions by Sause [15]. The simulated AE waveforms were compared with those obtained by analytical source functions and the best agreement was found for the ‘cosine bell’ function [16]. Le Gall et al. [17] also proposed a monopole point source in FE model to simulate AE signals from H-N sources. The FE model was validated and the influence of different AE sources, specimen geometry and piezoelectric sensor on the AE wave were investigated. Recently, AE waves induced from H-N sources were simulated by Cheng et al. [18] using excitation signals modulated as tone burst with a central frequency of 150 kHz at the FE model. The artificial neural network models were trained using the data collected in FE modelling and shown to predict AE source locations with satisfactory accuracy on a steel–concrete composite girder. Although FE modelling of AE signals from H-N sources has been widely studied over the past 20 years, the authors were unable to source any studies where FE modelled training data were used for delta-T mapping in the literature.

In this study, a numerical method for simulation of an H-N source was validated and applied on a complex plate to train a delta-T map for localization of experimental H-N sources. Results of the present investigation indicated that the FE generated delta-T map decreases the time and effort required for manually collecting and processing the training data, whilst maintaining a reasonable degree of source location accuracy. To the best of our knowledge, this is the first paper in which FE method was used to collect data to train delta-T maps. The FE generated delta-T mapping technique can be applied on monitoring active defects in complex structures as an accurate, low-cost and time-efficient method. The paper is arranged as follows: The numerical method for simulation of an H-N source is described and validated on a simple structure. Then, the delta-t map generated by the FE method is compared with the map from the experiment and traditional TOA techniques in a complex plate. Finally, findings are discussed and concluded in the last section.

## 2. Numerical Method for Simulating H-N Sources and Its Validation

A numerical method in which a monopole point force pulse acts in the out-of-plane direction was used to model an H-N source. Before applying this approach on a complex plate model, it was used to simulate an H-N source on a simple plate and numerical results were compared with the experimental results to validate the FE model.

### 2.1. Numerical Method for Simulating H-N Sources

**Figure 1:**
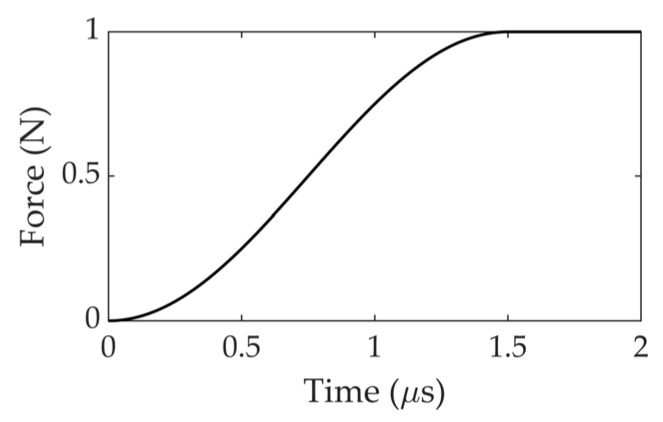
‘Cosine bell’ source characteristic.

Using the break of a pencil lead to generate acoustic waves, the H-N source [19,20,21] creates a step-function from a maximum compressive contact force and to 0 value in a very short time period [22]. A broadband range of frequencies is excited by the step-function force, which has been used for the primary sensor calibration in codes and standards [23]. Therefore, a commonly used step-function, ‘cosine bell’ function, which was proposed by Hamstad [16], was selected to simulate the time-dependent surface deflection on the plate caused by H-N source. The ‘cosine bell’ force pulse is described in Equation (1). The characteristics of ’cosine bell’ function sources are plotted and shown in Figure 1.

(1) $$F\left(t\right)=\{\begin{array}{cc} \;0\;N\;\;\;\;\;\;\;\;\;\;\;\;\;\;\;\;\;\;\; & ,t\leq 0\;\;\;\;\;\;\;\;\;\;\;\;\\ 0.5-0.5\times \cos\left(\frac{\pi \times t}{T}\right)\;N & ,0<t<T\;\;\;\;\;\;\;\;\\ 1\;N\;\;\;\;\;\;\;\;\;\;\;\;\;\;\;\;\;\; & \;,t>T\;\left(T=1.5\;\mu s\right) \end{array}$$

The pulse was applied to a point on the top surface of the FE model in the out-of-plane direction. As the actual load during a H-N source is unknown, the magnitude of the force was assumed to be 1 N [24]. As shown in Figure 1, the force pulse ramps up from 0 N to 1 N over a time duration of 1.5 µs, with a temporal resolution of 0.1 µs and then remains constant.

### 2.2. FE Model for Simulating an H-N Source on a Simple Plate

**Figure 2:**
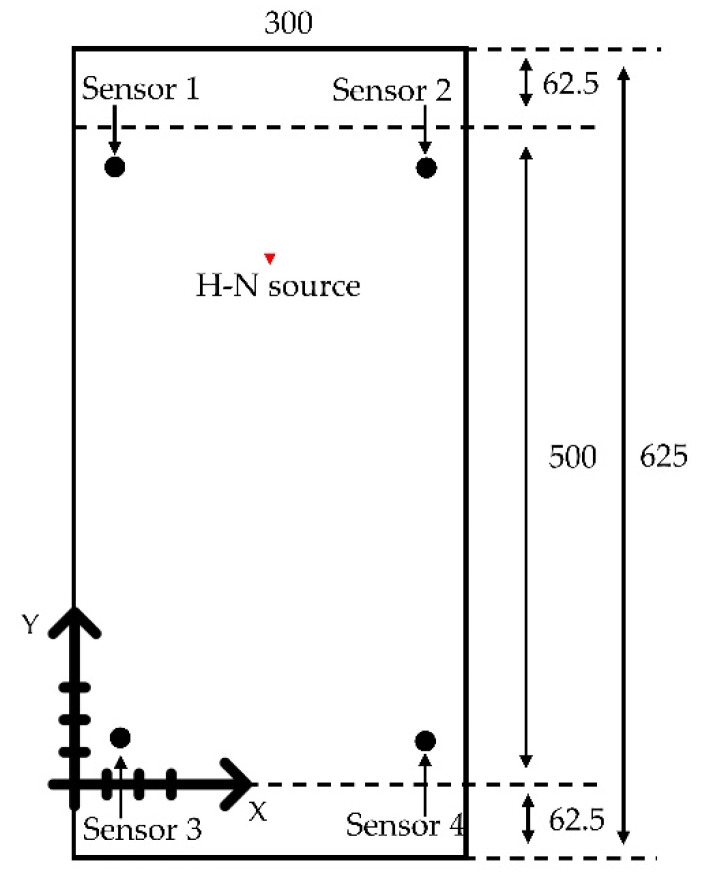
Schematic layout of the sensors and H-N source (units: mm).

To test the effectiveness of the numerical method described in Section 2.1, a simple plate model was created using a commercial code ABAQUS (Dassault Systèmes Simulia Corp., Providence, RI, USA). This model has a width of 300 mm in the X direction, length 625 mm in the Y direction and thickness 3 mm in the Z direction. Material properties of the steel plate are a density value of 7850 kg/m^3^, Young’s modulus of 210 GPa, and Poisson’s ratio of 0.3. A schematic layout of the sensors and the simulated H-N source is shown in Figure 2. The point force pulse described in Section 2.1 was excited at the location of the simulated H-N source. For the sake of simplicity and saving computing time [18], it was decided not to model an actual sensor and to only consider the out-of-plane displacement since the focus of the work was to model the delta-T mapping process rather than to model a sensor. A single node acted as the basis of the output signal; displacements in Z direction (out-of-plane) over time of all nodes at locations of sensors were used for the analysis. The central 500 mm section of the specimen was considered as test area in the investigation for the purpose of minimizing the influence of the edge reflections on the recorded waves. A coordinate system was introduced, and the origin was placed on the bottom left corner of the test area. The coordinates relative to the origin of four sensors and the H-N source are summarized in Table 1. The four outermost nodes at the bottom surface of the plate (Z = 0 mm) were fixed in X, Y, Z direction (Ux = Uy = Uz = 0) to prevent rigid body motion.

The convergence of numerical results is strictly dependent upon the temporal and the spatial resolution used in the FE model. To avoid numerical instability and enhance wave propagation accuracy, the shortest wavelength of the simulated elastic waves needs to be resolved by the mesh resolution [25,26,27].

According to the wavelength formula, the relationship between wavelength  λ and frequency  f is described by the following simple equation:

(2) λ=υ/f

where  υ is the wave velocity.

**Figure 3:**
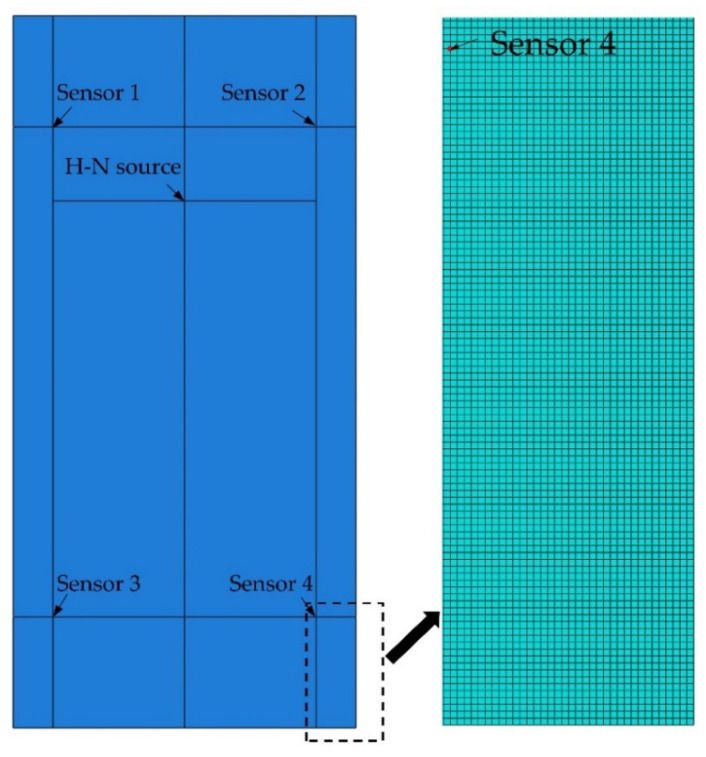
FE model and mesh in ABAQUS.

The maximum frequency of the wave was set as 500 kHz to align with the frequency range (100–450 kHz) of VS150-RIC (Vallen Systeme GmbH, Wolfratshausen, Germany) sensors used in the following experiments. Assuming the longitudinal and shear wave velocities in steel are 5940 m/s and 3220 m/s, respectively [28], the smallest wavelength, thereby the maximum mesh size for 500 kHz frequency was calculated to be 6.44 mm. As reported by Le Gall et al. [17], at least 5 nodes per wavelength are sufficient to simulate AE waves accurately with frequencies in the range of 500 kHz. Therefore, the mesh resolution of 1 mm, which gives 6 nodes per wavelength, was used for the simulations for both the accuracy and computational efficiency. The mesh convergence test also showed that 1 mm was sufficient mesh resolution for accurate simulation of elastic wave propagation. The general purpose 8-node linear brick element, C3D8R, provided by ABAQUS was used for the FE model. The FE model and mesh are shown in Figure 3.

Choosing an appropriate time integration step is also critical to achieve numerical stability and resolve the high frequency components accurately. An equation shows the relationship between time increment Δt and maximum of frequency $f_{max}$ was recommended by Moser et al. [29] and expressed as:

(3) $$\Delta t<1/\left(20f_{max}\right)$$

Given that the highest frequency of interest is 500 kHz, a maximum time integration step of 0.1 μs was calculated. In order to save computational time while maintain a sufficient a temporal resolution, a time integration step was set as 0.01 μs in accordance to the reference [15]. The time increment equalled to a sampling rate of 100 MHz, which did not correspond to the sample rate of the experimental tests (5 MHz). However, it should be noted that both the sampling rates used in the modelling and experiments are sufficient to resolve the signals’ highest frequency content of interest (500 kHz). Therefore, the error on time estimation between experiments and modelling caused by different sampling rates was marginal.

### 2.3. Experimental Verification of FE Model for Simulating an H-N Source on a Simple Plate

**Figure 4:**
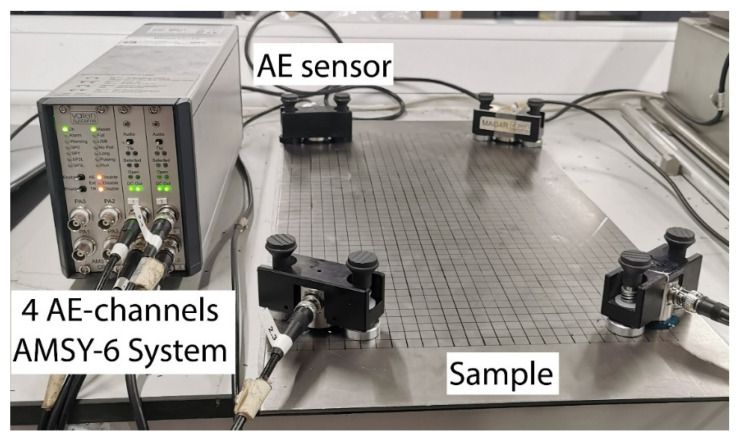
AE sensors mounted on 10 × 10 mm spaced grid specimen and connected to the AE systems.

In parallel, experimental testing of H-N sources on a simple mild steel plate was performed to validate the FE model. The dimensions of this plate were the same as those of the FE model in Section 2.2, 625 mm × 300 mm × 3 mm. The setup of the experiment is shown in Figure 4. In order to easily specify the locations of AE sensors and H-N sources on the plate, a grid with 10 mm spacing was drawn on the central 500 mm section of the specimen. A coordinate system identical to that of FE model was introduced. The coordinates relative to the origin of four sensors and the H-N source are the same as those presented in Table 1.

The AE waveforms were measured by four VS150-RIC sensors, which were acoustically coupled using ultrasound gel and pressure was applied using magnetic clamps. The integral pre-amplifiers with 34 dB gain amplified the signals recorded by sensors and outputs of the transducers were linked to a Vallen 4-channel AMSY-6 System. The signals were acquired using the AE system with the acquisition settings shown in Table 2.

The experiment was conducted in the lab at TWI Ltd., Cambridge, UK. Bubble wrap was used to acoustically de-couple the sample from the workbench surface. Before starting the experiment, an H-N source was generated close to each AE sensor to assess the sensor sensitivity and the coupling between the sensors and testing plate. Hits with amplitude above 98 dB at each sensor were recorded, which verified the coupling. A total of three H-N sources with 0.5 mm 2H type pencils were performed on the location of H-N source given in Table 1 to reduce erroneous data and provide a reliable average result. The amplitude remained constant with discrepancies within a couple of dBs for each sensor.

#### 2.3.1. Modal Analysis

When two surfaces are introduced in a medium (i.e., a plate-like structure), the longitudinal and transverse waves couple at the surfaces and form new surface waves known as Lamb waves. Lamb wave propagation is very complex. Velocities of Lamb wave modes have a strong dependency on frequency content and plate thickness. For a specific geometry, the relationship between wave velocity and frequency of each mode can be described by a set of curves known as dispersion curves. Two major Lamb waves are symmetric zero-order (S_0_) and antisymmetric zero-order (A_0_) modes.

Given the dispersive properties of elastic waves in flat plates, each wave mode with different frequencies has varied phase and group velocities, which results in the overlapping of some modes. Wavelet Transform (WT) is one of the most useful analysis tools for transient signals. It can be used to plot a time-domain graph showing the magnitude of the frequency spectrum of the signal. Hence, the amplitude of the AE waveform in both time and frequency domains can be viewed simultaneously. By overlapping the calculated dispersion curves with WT, the oncoming wave modes can be clearly distinguished.

**Figure 5:**
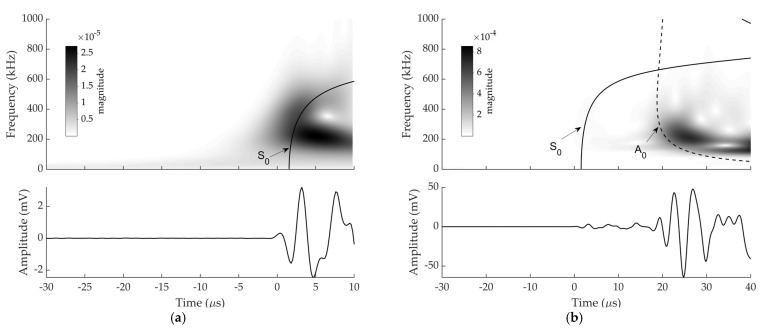
Experimental signal at sensor 1 and corresponding WT diagram with superimposed dispersion curves from: (**a**) −30 to 10 µs (scale was altered to make the S_0_ visible); (**b**) −30 to 40 µs.

**Figure 6:**
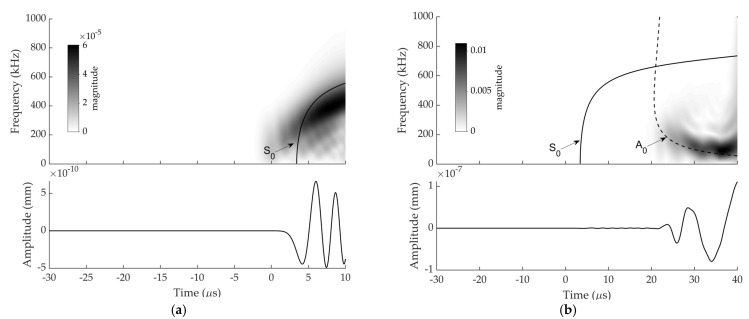
Numerical signal at sensor 1 and corresponding WT diagram with superimposed dispersion curves from: (**a**) −30 to 10 µs (scale was altered to make the S_0_ visible); (**b**) −30 to 40 µs.

Numerical and experimental signals at sensor 1, which were induced by an H-N source whose location was given in Table 1, are presented with WT diagrams processed using a Gabor wavelet in Figure 5 and Figure 6 respectively. Dispersion curves for Lamb wave propagation in a 3 mm steel plate were superimposed onto the WT diagrams. These have been shifted manually to achieve a best fit between the arrival of the fastest S_0_ mode in dispersion curve with the arrival of S_0_ mode on the WT colour plot.

The movements of particles are mainly parallel to the plate for S_0_ mode and perpendicular to the plate for the A_0_ mode [30]. When the H-N source is conducted in plane to the sensor face, a large portion of the resulting displacement is normal to the plane of the plate giving rise to A_0_ mode, while there is still a small portion of displacement tangential to the plate due to the Poisson effect, giving rise to the S_0_ mode. Consequently, A_0_ modes have a substantially larger out-of-plane amplitude than S_0_ modes [31]. WT for each signal were analysed under two time periods (i.e., before A_0_ arrival and after A_0_ arrival).

As can be seen in Figure 5 and Figure 6, S_0_ and A_0_ are discernible within both experimental and numerical signals even though S_0_ and A_0_ components were not well separated in the responses of the sensor 1. The S_0_ components have a faster velocity than the A_0_ components [32]. It is shown that the arrival of low amplitude S_0_ components are followed by high amplitude A_0_ component. The first-arrival waves were S_0_ modes with low amplitude, which were followed by the A_0_ modes with very high amplitude as expected.

It can be observed that the experimental and numerical WT diagrams show a reasonably good agreement. The discrepancy between the experimental and numerical WT magnitude at sensor 1 can be explained by the frequency response of the actual sensors not being simulated in the FE modelling.

#### 2.3.2. Arrival Time Estimation

Determination of arrival time of signals is crucial for localization of the AE source. Three arrival time estimation methods, which are threshold crossing, the Akaike Information Criterion (AIC) and WT analysis are discussed in the following.

The default time arrival estimation method in commercially available AE systems is threshold crossing. In this method, the arrival timing will be triggered, and an AE hit will be recorded only when a signal crosses a pre-set threshold level. Because of factors including the assumptions on the magnitude of the force, the time duration during an H-N source and a simplified sensor model, accurate prediction of AE signals’ amplitudes using the FE model were not achieved and not within the scope of this work. Moreover, displacement was measured in FE model while voltage was measured in the experiment. Consequently, a same threshold level cannot be found for both experimental and numerical signals in the threshold-crossing method. Therefore, this method was only used to estimate arrival times of experimental signals.

**Figure 7:**
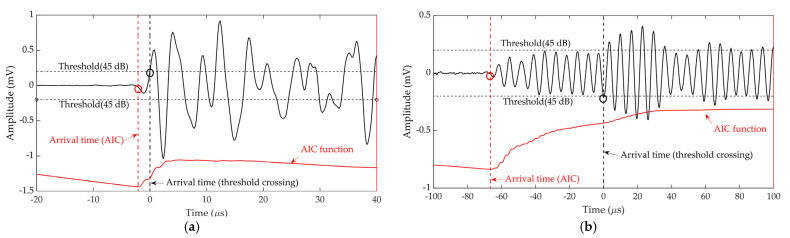
An example of arrival time estimation using AIC and threshold crossing on (**a**) high amplitude and (**b**) low amplitude AE signals.

For the threshold-crossing method, it is difficult to choose the appropriate threshold level. Triggering errors might come from missing the start of the wave as shown in Figure 7b. A reduced threshold can improve the arrival time measurement accuracy [6]. However, background noise bearing a lower amplitude than the pre-set threshold can falsely trigger AE acquisition, resulting in erroneous arrival time. Therefore, in addition to threshold-crossing method, AIC was also used in this study to estimate the arrival times of AE signals. The AIC function [33] compares the difference in classic variance before and after each point in the wave, typically when it is at its minimum represents the point where the transition from noise to waveform is occurring, and therefore the arrival time of the wave. It can be observed that the threshold-crossing error was around 2 μs on a high-amplitude signal from an H-N source in Figure 7a, while it was around 65 μs on a low-amplitude signal from a corrosion test in Figure 7b which is presented for demonstration. However, the AIC method detected the accurate onset time of signal regardless of changes to the amplitude. Moreover, the robust performance of AIC function to pick the arrival times of signals with varied signal-to-noise ratios was demonstrated by Kurz et al. [33] and Pearson et al. [6]. Therefore, the AIC method has been demonstrated to be a reliable onset time determination tool.

The arrival times can also be predicted by considering the peak WT magnitudes at a mode known frequency, as shown by Yamada et al. [34], Hamstad et al. [35] and Jeong and Jang [36]. It was demonstrated that the maximum magnitude of WT in the time-frequency domain corresponds to the arrival time of mode wave traveling with group velocity. Hence, the arrival times of specific frequency component can be determined. In this study, different dominant Lamb wave modes over time were shown using WT in Section 2.3.1. The frequency component of 200 kHz was used in the WT analysis to determine the arrival time of mode waves. This frequency was chosen because S_0_ modes can be observed at this frequency according to the spectral analysis of captured signals and the peak wavelet coefficients of S_0_ modes are conspicuous at this frequency. The arrival time of S_0_ modes at 200 kHz was determined based on the peaks of the WT coefficients at 200 kHz.

In general, threshold crossing was only used to estimate the arrival time of experimental signals induced by an H-N source on a simple plate and AIC and WT analysis were used on both experimental and numerical signals. For simplicity, delta-T for sensor 1 and sensor 3 was calculated. Results are summarized in Table 3.

The estimated delta-T produced by threshold crossing, AIC and WT analysis in the experiment showed very little difference. Compared with delta-T determined by AIC, threshold crossing and WT analysis showed a discrepancy of 1.6 µs and 0.4 µs, respectively. The simulation predictions appeared to be well in agreement with experimental observations. For the delta-T estimated by AIC, a 0.06 µs discrepancy was given between the experiment and FE modelling. Good agreement was also demonstrated between delta-T in the experiment predicted by WT analysis and that of modelling, with a discrepancy of 0.63 µs. Moreover, for the simulation results, delta-T given by WT analysis was very close to that given by AIC.

Overall, experimental and numerical WT diagrams show a reasonably good agreement. A high level of accuracy of the delta-T from FE modelling can be observed. It can thus be concluded that a numerical method for simulation of an H-N source on a simple plate was validated. The validation of this numerical method demonstrated great potential on making it a useful tool for predicting arrival times of AE waveforms induced by H-N sources on a complex FE plate model. For arrival time estimation, results show that AIC represents a viable option for estimating the onset of signals. As the accuracy of threshold crossing depends on the pre-set threshold level, and the arrival time determined by WT analysis is the time at a single frequency, AIC was used to estimate arrival time of signals in numerical and experimental delta-T techniques.

## 3. Experimental and Numerical Delta-T Mapping Training on Complex Plate

Using the validated numerical method described in Section 2.1, H-N sources were simulated on a complex plate model. Information including arrival times of signals at each sensor was collected to build the numerical delta-T map. In parallel, experimental delta-T mapping was also calculated on a complex plate for the localization of experimental AE events. Localization results produced by numerical and experimental delta-T mapping were compared.

### 3.1. Experimental Delta-T Mapping Training on Complex Plate

**Figure 8:**
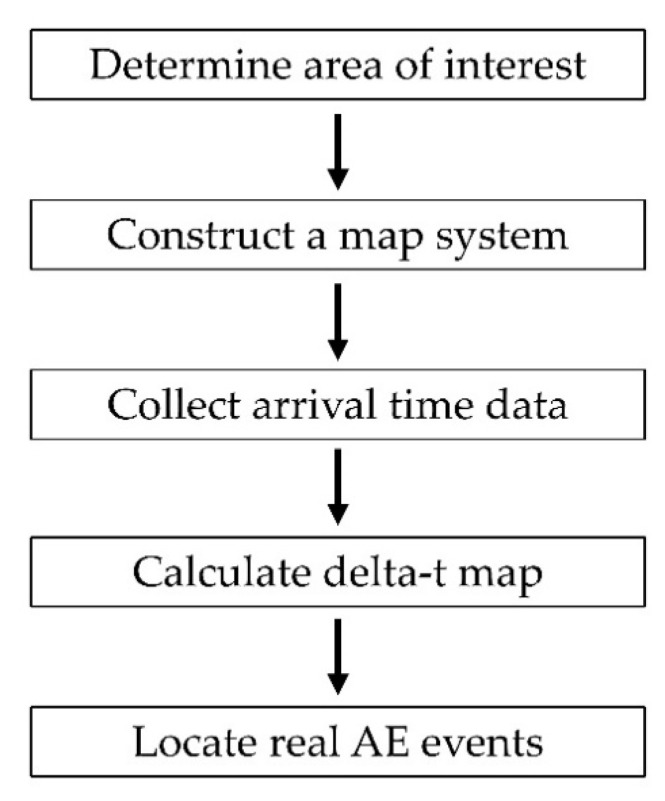
Procedural steps of implementing delta-T mapping technique [5].

The delta-T mapping technique requires the structure to be mapped whereby test data are compared to the training maps which are created by performing H-N sources and evaluating the difference of arrival times for each sensor pair to estimate the source location. The procedural steps of implementing the delta-t mapping technique [5] are presented in Figure 8. By performing H-N sources on each node of the grid, factors including complex geometric features can be considered within mapping area and detailed accurate wave speed data are not required. Therefore, this technique shows its advantage on source location in complex structures.

**Figure 9:**
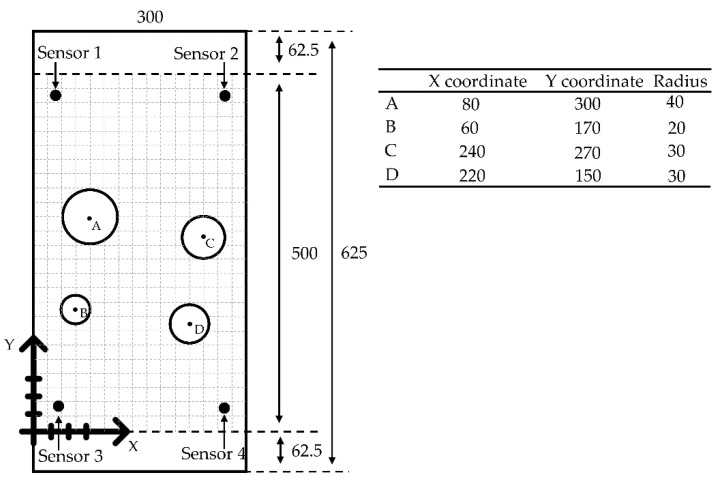
Schematic layout of the sensors on a complex plate model with 20 mm spacing grid (unit: mm).

A complex geometry mild steel plate with four holes was used for experimental delta-T mapping training. The dimensions of the specimen with coordinates of the centres of four holes and radiuses are shown in Figure 9. The locations of all the sensors were the same as those in the experiment on simple plate described in Section 2.3. Following procedural steps shown in Figure 8, the central 500 mm section was determined as the area of interest and a coordinate system was introduced. After that, a training grid was placed on this area. The spacing of the grid was determined to be 20 mm as prior research [6] has shown that this is the point at which further reduction in size has minimal influence on accuracy. The arrival time data, which were determined by AIC, were collected after the generation of an H-N source at each node of the grids. For each sensor pair, a delta-T map was produced. The number of maps produced will depend on the number of sensors given by the equation  N*(N−1)/2, where N is the number of sensors in the network. By comparing the delta-T for an unknown source with those of the maps, the location can be determined. It should be noted that there is a significant interaction of waves with holes and plate edges. However, the basis of the delta-T mapping is the first arrival time of signals, and this interaction is beyond the scope of this study.

### 3.2. FE Generated Delta-T Mapping Training on Complex Plate

**Figure 10:**
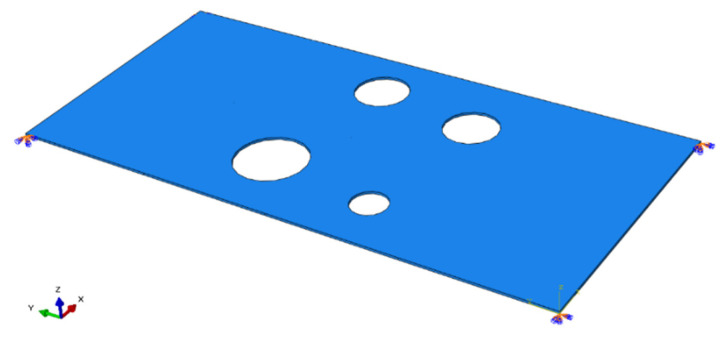
Complex geometry plate modelled in ABAQUS.

In parallel, FE modelling was performed to produce a FE generated delta-T map or numerical delta-T map. As shown in Figure 10, a complex plate model same as the plate in Section 3.1 was created in ABAQUS. As with the FE modelling of H-N sources on simple plate in Section 2.2, steel material properties, mesh resolution of 1 mm, time integration step of 0.01 μs, C3D8R element and same boundary conditions were used for simulation. The locations of sensors and the grid were chosen to be the same as those of the experiment on complex plate in Section 3.1. The validated numerical method described in Section 2.1 was used to simulate an H-N source on each node of the grid. Displacements in Z direction (out-of-plane) over time of all nodes at locations of sensors were recorded in FE modelling to estimate the arrival time. AIC was used to determine the arrival times of simulated signals. The FE generated delta-T map was built in the same way as experimental delta-T map in Section 3.1.

### 3.3. Experimental Test Data on Complex Plate

In order to test the performance of experimental and numerical delta-T maps, six extra H-N sources (H-N source 1, 2, 3, 4, 5 and 6) were conducted at six off grid locations on complex plate in the experiment. The coordinates of the locations of six extra H-N sources relative to the origin are given in Table 4.

### 3.4. Results

**Figure 11:**
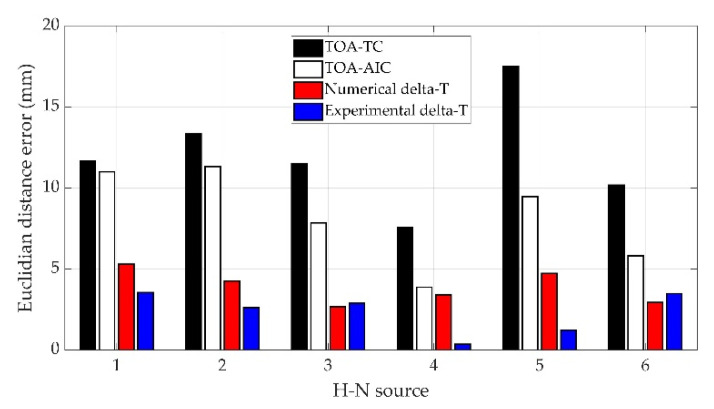
Euclidian distance errors of source location results.

**Figure 12:**
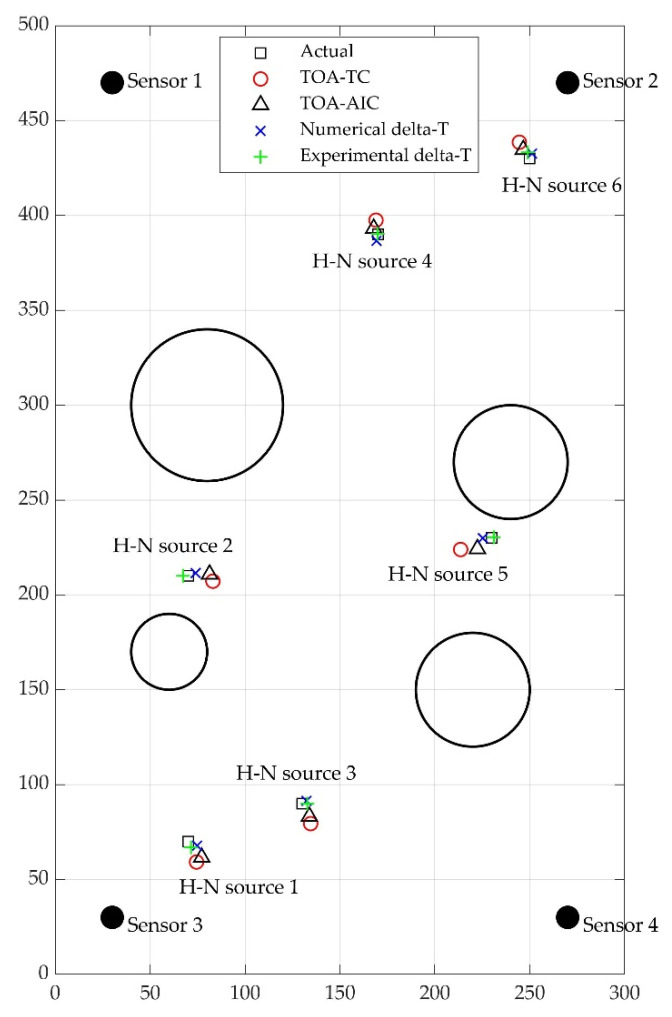
Actual locations of H-N sources and results calculated by TOA-TC, TOA-AIC, numerical delta-T and experimental delta-T (units: mm).

The AE test data collected from H-N source 1, 2, 3, 4, 5 and 6 were assessed with the traditional TOA location approach based on threshold crossing (or TOA-TC for short), TOA location approach based on AIC method (or TOA-AIC), experimental delta-T mapping (or experimental delta-T) and numerical delta-T mapping (or numerical delta-T) techniques. Table 4, Figure 11 and Figure 12 documented source location results and the associated Euclidian distance errors.

For H-N source 1 and 2, it can be observed that a small improvement in accuracy was observed after the AIC method was used in the TOA localization technique. The errors were reduced significantly using the numerical delta-T and experimental delta-T respectively compared with that of the TOA-TC. For H-N source 3 and 6, the TOA-AIC produced location errors of 7.84 mm and 5.81 mm compared with 11.51 mm and 10.18 mm calculated by TOA-TC, respectively. Results at H-N source 3 and 6 showed that the experimental and numerical training approaches produced similar levels of error, with a slightly higher accuracy given by numerical training approach. For H-N source 4, TOA-TC had a location error of 7.58 mm compared with 3.87 mm using TOA-AIC, a 49% reduction using TOA-AIC. The results showed little difference on location error when TOA-AIC and numerical delta-T were used, with numerical delta-T having slightly increased accuracy. However, the accuracy of source location was significantly better using experimental delta-T with a Euclidean distance error of 0.36 mm. For H-N source 5, the TOA-TC, TOA-AIC and numerical delta-T located the H-N source slightly to the left of the actual position with a Euclidean error distance of around 17.49 mm, 9.47 mm and 4.73 mm, respectively, while experimental delta-T located the source to the right of the actual location with an error of 1.22 mm. Experimental delta-T and numerical delta-T showed an obvious improvement over the TOA-TC and TOA-AIC. The average errors of H-N source locations calculated by experimental and numerical delta-T are 2.35 mm and 3.88 mm, respectively.

In general, the TOA-TC technique produced highest location errors because of indirect wave path and unreliable arrival time estimation method. Since the AIC method is considered to be more accurate in arrival time determination than threshold crossing [6], TOA-AIC led to a marked improvement in accuracy. As had been discovered in previous works [1,6,8,9,37,38], the delta-T mapping technique improved the source location accuracy on the complex plate. The presented results show the noticeable improvement in source location accuracy using experimental delta-T and numerical delta-T methods over TOA-TC and TOA-AIC. Because a coarse training grid was used in experimental delta-T and numerical delta-T, the accuracy of the location results may be further improved with a higher spatial resolution of the training grid. Other than H-N source 3 and 6, experimental delta-T shows a higher degree of accuracy over numerical delta-T, which can be explained by errors of FE analysis in numerical delta-T including the modelling errors associated with the simplification and mesh discretization error. It is also worth noting that the locations of the artificial damage were calculated with sufficient accuracy by both experimental delta-T and numerical delta-T.

## 4. Discussions

The FE generated or numerical delta-T mapping techniques offer certain advantages over experimental delta-T. Firstly, for experimental delta-T, it requires several days to set up the experiment, perform H-N sources on all the grid points, select and prepare the AE data to build the delta-T maps. For the numerical delta-T, it takes 0.5 days to build an FE model. The computation time for simulation of an H-N source on each node of the grid is 5 min on a personal laptop. There is a total of 285 nodes in the present model, which results in a computation time of 1 day. The total time required for the whole process is 1.5 days. This reveals that the numerical delta-T decreases the time required for constructing a delta-T map. It is worth noting that processes of experimental and numerical delta-T mapping could be optimised to reduce these times. To ensure maximum accuracy, an operator with an AE background is required to manually sort and discard useless arrival time data being captured experimentally [38]. With numerical delta-T, data are captured autonomously, which means that collection and processing of training data do not rely on experience and human errors are removed. Moreover, the expenses associated with the equipment and labour required for experiments can be eliminated. Furthermore, the numerical delta-T mapping method scales better compared with experimental delta-T. With the FE method, large-scale models with numerical sensors can be easily created for collection of training data, whilst experimental collection of delta-T data may be limited by several factors including large size or complex structures. Moreover, instead of artificial AE sources which can only be performed on the surface in experiments, the FE method allows multiple training sources such as fatigue [39] and fibre breaks [40] and internal damage to be modelled. This might provide a solution to a 3-D delta-T location problem. A final benefit of numerical delta-T is that the risk of foreign object debris from broken pencil leads is eliminated, which in some sectors is a major consideration.

However, numerical delta-T also bears some disadvantages. One of the main difficulties lies in the modelling of wave propagation in highly complex structures, such as composites which are being used abundantly in safety critical structures in recent years. Furthermore, fine mesh is required to model AE waves with high accuracy. The number of degrees of freedom in a large complex FE model will be huge, leading to a significant demand for computational resources such as memory space and processor time. The solution for this could be the use of fine meshes over the area of interest and coarse meshes over other areas.

Although the FE generated delta-T was carried out successfully, there are some limitations to this study. Firstly, one such limitation is that TOA-TC, TOA-AIC, numerical delta-T and experimental delta-T have not been trialled to locate real AE data. As real damage sources [12] usually exhibit a smaller amplitude and different frequency content compared with H-N sources, it will be of interest to examine the performance of four methods with AE signals generated from real damage mechanisms. Secondly, only holes were considered in the complex plate in the present study; however, other complexities of real structures including multiple thickness changes, stiffeners, holes, nozzles, welds, etc., need to be considered in future study. Thirdly, the defined boundary conditions in the model were not the same as real boundary conditions of the plate. The wave propagation will be slightly influenced by the edge of the hole on the complex plate and thus might result in errors in arrival time. By applying realistic boundary conditions in the FE model, the influence of the edges of holes on the first arrival of AE waves can be eliminated. The accuracy of arrival time of signals recorded in complex FE model can be improved. Moreover, another source of error is that the AE sensor face is not considered, as the sensor is simplified as a point in the modelling. It could be stated that the signal in experimental training is captured at the sensors’ circumference rather than sensors’ centre point where simulated sensors are located, thereby resulting in different arrival times. A numerical study conducted by Tsangouri and Aggelis [41] showed that the size of the simulated AE sensor bears a direct effect on wave content in time domains. The source location accuracy produced by finite element generated delta-T maps may be improved if the AE sensor with the sensor face can be modelled. Furthermore, the material defined in the FE model was an isotropic homogenous metallic material which was considered in this investigation. As composites are being increasingly used in the oil and gas industry, it would be beneficial to examine the performance of a modelled delta-T training dataset on composite materials. Moreover, it should be noted that the delta-T mapping technique and other localization techniques do not account for factors such as temperature, loading and sensor coupling changes. It will be of interest to investigate how these factors will influence the delta-T maps and identifying the methods to update delta-T maps accordingly. Finally, current commercial AE systems remain incapable of the live application of experimental and numerical delta-T mapping techniques. However, it will be beneficial to integrate these methods with commercial AE systems to observe online AE events.

## 5. Conclusions

In this investigation, a numerical method from the literature was used to simulate an H-N source on a simple plate and was validated by the experiment results. The validation of the FE model exhibited great promise for application of the FE method to a planar location problem on a complex plate. Using the same numerical method, H-N sources were simulated on a complex plate and a numerical delta-T map was generated. The location results of the numerical delta-t map technique were compared with those of traditional TOA techniques and an experimental delta-t map technique. The viability of using the FE method was demonstrated to decrease the time and labour required for manually collecting and processing training data whilst maintaining a reasonable degree of source location accuracy with an average error of 3.88 mm. With such a high source location accuracy, the specific area of concern requiring inspection using other NDE techniques can be greatly reduced. Moreover, if the specific location of an AE event is identified on an area with certain geometric features or loading conditions, the number of potential damage source mechanisms can be decreased.

Although the FE generated delta-T mapping was conducted successfully, it would be preferable to examine the performance of present FE generated delta-T maps with AE signals from real damage mechanisms. Development of the FE model such as improved boundary conditions, realistic AE sensors, additional complexity and anisotropic materials requires further investigation.

## Figures and Tables

**Table 1:** Coordinates of sensors and H-N source (units: mm).

	X Coordinate	Y Coordinate
Sensor 1	35	465
Sensor 2	265	465
Sensor 3	35	35
Sensor 4	265	35
H-N source	150	400

**Table 2:** AE acquisition settings.

Threshold (dB)	Sample Length (ms)	Sample Rate (MHz)	Pre-Trigger (ms)	Rearm Time (ms)	Duration Discrimination Time (ms)
45	1.6	5	0.1	0.8	0.8

**Table 3:** Delta-T for sensor 1 and sensor 3 in the experiment and FE modelling (units: µs).

	Threshold Crossing	AIC	WT Analysis
Experiment	48.4	46.8	47.2
FE modelling	-	46.74	46.57
Difference	-	0.06	0.63

**Table 4:** Actual locations of H-N sources and results calculated by TOA-TC, TOA-AIC, numerical delta-T and experimental delta-T (units: mm).

	H-N Source 1			H-N Source 2			H-N Source 3			H-N Source 4			H-N Source 5			H-N Source 6		
	X	Y	Error	X	Y	Error	X	Y	Error	X	Y	Error	X	Y	Error	X	Y	Error
Actual	70	70	-	70	210	-	130	90	-	170	390	-	230	230	-	250	430	-
TOA-TC	74.42	59.19	11.68	83.04	207.21	13.34	134.54	79.42	11.51	168.99	397.51	7.58	213.60	223.92	17.49	244.54	438.59	10.18
TOA-AIC	77.13	61.63	11.00	81.29	210.88	11.32	133.89	83.19	7.84	167.79	393.18	3.87	222.44	224.29	9.47	246.58	434.70	5.81
Numerical delta-T	74.82	67.80	5.30	73.99	211.46	4.25	132.26	91.41	2.66	169.33	386.67	3.40	225.27	229.86	4.73	251.17	432.7	2.94
Experimental delta-T	71.54	66.80	3.55	67.39	210.13	2.61	132.89	90.03	2.89	169.93	390.35	0.36	231.13	230.45	1.22	248.96	433.33	3.49

## Data Availability

The data presented in this study are available on request from the corresponding author.
